# No substantial changes in estrogen receptor and estrogen-related receptor orthologue gene transcription in *Marisa cornuarietis* exposed to estrogenic chemicals^☆^^☆☆^

**DOI:** 10.1016/j.aquatox.2013.05.002

**Published:** 2013-09-15

**Authors:** Richard Bannister, Nicola Beresford, David W. Granger, Nadine A. Pounds, Mariann Rand-Weaver, Roger White, Susan Jobling, Edwin J. Routledge

**Affiliations:** a Brunel University London Institute for the Environment, Uxbridge, Middlesex UB8 3PH, UK; b School of Health Sciences and Social Care, Brunel University, Uxbridge, Middlesex UB8 3PH, UK; c Imperial College London, Institute of Reproductive & Developmental Biology, London, England, UK; d AstraZeneca Safety, Health & Environment, Brixham Environmental Laboratory, Freshwater Quarry, Brixham, Devon TQ5 8BA, UK

**Keywords:** Mollusc, Estrogen receptor, Estrogen-related receptor, Gene transcription, Estrogen, Exposure

## Abstract

Estrogen receptor orthologues in molluscs may be targets for endocrine disruptors, although mechanistic evidence is lacking. Molluscs are reported to be highly susceptible to effects caused by very low concentrations of environmental estrogens which, if substantiated, would have a major impact on the risk assessment of many chemicals. The present paper describes the most thorough evaluation to-date of the susceptibility of *Marisa cornuarietis* ER and ERR gene transcription to modulation by vertebrate estrogens *in vivo* and *in vitro*. We investigated the effects of estradiol-17β and 4-*tert*-Octylphenol exposure on *in vivo* estrogen receptor (ER) and estrogen-related receptor (ERR) gene transcription in the reproductive and neural tissues of the gastropod snail *M. cornuarietis* over a 12-week period. There was no significant effect (*p* > 0.05) of treatment on gene transcription levels between exposed and non-exposed snails. Absence of a direct interaction of estradiol-17β and 4-*tert*-Octylphenol with mollusc ER and ERR protein was also supported by *in vitro* studies in transfected HEK-293 cells. Additional *in vitro* studies with a selection of other potential ligands (including methyl-testosterone, 17α-ethinylestradiol, 4-hydroxytamoxifen, diethylstilbestrol, cyproterone acetate and ICI182780) showed no interaction when tested using this assay. In repeated *in vitro* tests, however, genistein (with mcER-like) and bisphenol-A (with mcERR) increased reporter gene expression at high concentrations only (>10^−6^ M for Gen and >10^−5^ M for BPA, respectively). Like vertebrate estrogen receptors, the mollusc ER protein bound to the consensus vertebrate estrogen-response element (ERE). Together, these data provide no substantial evidence that mcER-like and mcERR activation and transcript levels in tissues are modulated by the vertebrate estrogen estradiol-17β or 4-*tert*-Octylphenol *in vivo*, or that other ligands of vertebrate ERs and ERRs (with the possible exception of genistein and bisphenol A, respectively) would do otherwise.

## Highlights

• ER and ERR transcription levels were unaffected in *Marisa cornuarietis* exposed to 17β-estradiol or 4-*tert*-Octylphenol.
• The mollusc ER protein interacts with the phytoestrogen genistein in transfected HEK-293 cells.
• The mollusc ERR protein interacts weakly with bisphenol-A in transfected HEK-293 cells.
• The mollusc ER protein binds to the vertebrate consensus estrogen response element (ERE) sequence.

## 1 Introduction

The susceptibility of molluscs to morphological and physiological disruption by estrogenic compounds is a subject of current debate. Amongst the most extreme effects reported are those exerted by bisphenol A (BPA), 4-*tert* octylphenol (4-*t*-OP) and 17α-ethinylestradiol (EE2) on reproductive output and morphology of the neo-tropical freshwater snail *Marisa cornuarietis* (Oehlmann et al., 2000, 2006; Schulte-Oehlmann et al., 2004). These published reports indicate that this species is extremely sensitive to very low concentrations of these compounds. The effects include increased oocyte production and egg-laying in females and gross morphological effects on the sex organs in both developing juveniles (*e.g.* formation of additional sex organs in females) and adults (*e.g.* reduction in male penis length). In direct conflict with these reports are those in which adult *M. cornuarietis* were exposed to BPA using a different experimental design to those employed in (Oehlmann et al., 2000) and (Oehlmann et al., 2006) showing clearly that these effects were not observed (Forbes et al., 2007). These conflicting reports have fuelled controversy (Dietrich et al., 2006) surrounding the true sensitivity of this species, and molluscs in general, to estrogen mimics and the perceived safety of the aquatic environment from the impacts of these xenoestrogens. BPA, in particular, is purported to be much more potent in molluscs than in other aquatic organisms (Oehlmann et al., 2006).

Perhaps the most striking feature of the effects reported (Oehlmann et al., 2000, 2006; Schulte-Oehlmann et al., 2004) is their resemblance to those that occur in vertebrate species in response to xenoestrogen exposure (Jobling et al., 2004). This may indicate that one or more estrogenic mechanisms have been conserved through evolution and are present in both molluscs and vertebrates. In vertebrates, the classically understood mechanism of estrogen action is *via* estrogen receptors (ERs). There are two main ER subtypes, ERα (Green et al., 1986) and ERβ (Kuiper et al., 1996). Lipophilic estrogens can enter cells *via* passive diffusion and are subsequently bound by ERs. This binding results in a conformational change in the ER protein, which then binds to specific DNA recognition sequences in the promoter regions of various genes and can regulate their transcription (Kimbrel and McDonnell, 2003). Published studies show that ER orthologues have been identified in several species of mollusc (Thornton et al., 2003; Keay et al., 2006; Kajiwara et al., 2006). However, all of these studies show lack of binding of ER protein to vertebrate estrogens or xenoestrogens *in vitro* using reporter assays, and endogenous ligands have not been found. Our laboratory published the cDNA cloning of a molluscan orthologue of vertebrate ERs from *M. cornuarietis* (Bannister et al., 2007). One of these factors, mcER-like, is highly similar to vertebrate ERs in terms of sequence similarity and structure; however, unlike its vertebrate orthologues (Routledge et al., 2000), it does not bind 17β-estradiol (E2). A second factor was cloned with high sequence identity to vertebrate (Giguere et al., 1988) and insect (Östberg et al., 2003) estrogen receptor-related receptors (ERRs). Like mcER-like and vertebrate and invertebrate ERRs, mcERR protein did not bind E2 in radio-ligand binding trials. Indirect evidence of ER-mediated estrogen binding is provided by Oehlmann and colleagues (Oehlmann et al., 2006), who observed that *in vivo* co-exposures of *M. cornuarietis* to BPA and classical anti-estrogens (Tamoxifen and Faslodex) negated the responses attributed to BPA. In the same study, it was shown that tritiated E2 could be displaced from cytosolic extracts of *M. cornuarietis* tissues by unlabelled E2. None of the above work demonstrates specific binding to, or activation of, classical estrogen-signalling pathway factors *in vivo*. This is necessary in order to prove that steroid estrogens and endocrine disrupting compounds interact with mollusc reproductive systems through interaction with the ER in *M. cornuarietis* and other species of mollusc. Here we test the hypothesis that the reported sensitivity of *M. cornuarietis* to estrogenic chemicals is mediated by the mollusc ER and/or ERR by examining the transcriptional responses of these genes during a 12-week exposure to a natural vertebrate steroid estrogen (E2) or a xenoestrogen (4-*t*-OP). We also assess the ability of 4-*t*-OP, E2 and a range of other natural and synthetic estrogenic chemicals (known to interact with estrogen receptors in vertebrates) for their ability to mediate reporter gene expression in a 2-hybrid system comprising HEK-293 cells transfected with mcER-like and mcERR ligand-binding domains coupled to the GAL4-DNA-binding domain and the VP16-transactivation domain.

## 2 Materials and methods

### 2.1 Chemicals

17β-Estradiol (E2; ≥98% pure), 17α-ethinylestradiol (EE2; ≥98% pure), genistein (GEN; ≥98%), diethylstilbestrol, (DES; ≥99%), bisphenol A (BPA; 97%), cyproterone acetate (CPA; ≥98%), methyl-testosterone (MT; ≥97%), hydroxy-tamoxifen (Tam-OH; ≥98%), ICI 182,780 (≥98%), and 4-*tert*-octylphenol (4-*t*-OP; 97%) were purchased from Sigma (Dorset, UK) and were research grade chemicals. Stocks of E2 and 4-*t*-OP for use in the *in vivo* exposure were prepared in methanol.

### 2.2 Animals and husbandry

*M. cornuarietis* were obtained from stocks bred at Brunel University, UK, which were originally derived from a stock maintained by Prof. Jörg Oehlmann's laboratory (Johann Wolfgang Goethe University, Frankfurt-am-Main, Department of Ecology & Evolution, Frankfurt-am-Main, Germany). Snails were maintained under static conditions in large 60 L static glass tanks at 22 °C and a photoperiod of 12l:12d and fed with organic lettuce. The exposures were carried out at AstraZeneca's Brixham Environmental Laboratory.

### 2.3 *In vivo* exposure to E2 and 4-t-OP experimental design

Three replicate groups of 20 adult snails between 6 and 8 months old (each containing 10 male and 10 female snails based on morphological examination) were exposed to dilution water control, a solvent control, E2 (at 10, 100 and 1000 ng/L) and 4-*t*-OP (5 and 25 μg/L) in flow through aquaria (volume 20 L) at 22 °C with a 12-h light/dark cycle and 0.025 g Tetramin flake food (Tetra) per snail per day (5 days per week). All flake food is tested for absence of estrogenicity using the yeast estrogen screen assay (Routledge and Sumpter, 1996) prior to use. Water samples were also taken for determination of E2 levels using the Yeast Estrogen Screen (Routledge and Sumpter, 1996) and 4-*t*-OP by HPLC analysis conducted at AstraZeneca. Samples were taken 1 week before exposure and throughout the 12 week exposure (approximately 3, 9 and 12 weeks after exposure) to ensure that the dosing into the water produced concentrations close to nominal values. Sampling of the snails (2 male and 2 females per tank selected at random to obtain 6 snails of each sex per treatment) took place 24 h prior to exposure, and after one week, six weeks and 12 weeks of exposure. Each snail was weighed and measured (shell height and aperture width) and tissues (ganglia, gonad-digestive complex, male penis and sheath, female albumin glands) were dissected out and snap frozen on liquid nitrogen for RNA extractions. Absolute quantitative real-time PCR (QPCR) was carried out to measure mcER-like and mcERR-like mRNA transcript abundance in each of the tissues in each snail as described previously (Bannister et al., 2007). Reproductive output in terms of numbers of eggs and egg-masses produced was recorded at 2-week intervals throughout the study (including the two-week baseline period).

### 2.4 Quantitative real-time PCR (QPCR)

Total RNA was isolated from individual organs of replicate individuals, using Tri-Reagent (Sigma), according to the manufacturer's recommended protocol. All total RNA samples were treated with *DNase* I (Invitrogen) to remove traces of genomic DNA. To determine absolute amounts of transcripts, RNA standards were synthesised *in vitro* from DNA templates, and Absolute Quantitative Real-time PCR was carried out using the one-step QuantiTect SYBR Green RT-PCR kit (Qiagen) with an ABI PRISM 7900 Sequence Detection System (Applied Biosystems) as described previously (Bannister et al., 2007). Each experiment included absolute negative controls (no template), no reverse transcriptase controls, negative tissue controls (RNA isolated from fathead minnow testis) and internal reference tissue controls (non-exposed *M. cornuarietis* RNA preparation). To account for inter-assay variability between different qPCR plates, a positive control (RNA prepared from non-exposed female *M. cornuarietis* albumin gland) was included in each assay plate. The qPCR data presented here are expressed as quantities relative to this internal control (using the formula *E*^(Ct*X* − Ct*Y*)^, where *E* is the efficiency of the qPCR reaction (calculated using the formula *E* = 10^(−1/slope)^, Ct is the threshold cycle, ‘*X*’ refers to the internal control and ‘*Y*’ refers to the sample (unknown). All determinations were carried out in triplicate.

### 2.5 Analytical chemistry

For E2 analysis all water samples (1 L) were passed through a C18 SPE column (Waters, UK) and the column dried completely by drawing through air. The columns were then immediately frozen and shipped to Brunel University (London, UK) for Yeast Estrogen Screen (YES) analysis. The SPE columns were eluted with analytical grade methanol and evaporated to dryness under a stream of nitrogen. The dried extract was then redissolved in absolute ethanol for analysis in the YES (Routledge and Sumpter, 1996) as described previously. The activity of water extracts was compared to a standard curve of E2 in order to establish the initial concentration of E2 in the exposure water (expressed as ng E2 equiv./L) and the average exposure over the course of the experiment.

Concentrations of 4-*t*-OP in the exposure tanks were measured by HPLC (150 mm × 4.6 mm Gemini 5 μm C18 column) using a fluorescence detector (272 nm and 310 nm excitation and emission wavelengths, respectively, bandwidth 40 nm, gain 100) and quantified by comparison against known standards of test substance (LOD was approximately 1.0 μg/L). Water sample aliquots were diluted with an equal volume of acetonitrile prior to analysis.

### 2.6 Electrophoretic mobility shift assays

The potential for the mcER-like protein to bind a consensus ^32^P-labelled estrogen response element (ERE) was investigated using an Electrophoretic Mobility Shift Assay (EMSA). All *in vitro* translations of the full length mollusc ER-like receptor (cloned into Stratagene pSG5 expression plasmids) were performed using a TNT^®^ coupled rabbit reticulocyte lysate system, with the appropriate RNA polymerase (T7), according to the manufacturer's instructions (Promega). The transcription/translation product from a reaction containing pSG5-mouseER (a gift from Prof. Malcolm Parker) was used as a positive control. Two μL of TNT reaction mixture total protein was added to 1 μL (equivalent to 10 ng) of ^32^P-labelled oligo (ERE) and incubated at room temperature for 20 min. Specific binding of the translated proteins to the labelled oligos was assessed by introducing unlabeled oligo in a 100-fold molar excess. Samples were run on 6% polyacrylamide gels at 250 V for 3 h and then visualised by autoradiography.

### 2.7 Ligand screening assays

PCR fragments generated from pSG5-GAL4-ER (containing the GAL4 DNA-binding domain (DBD) fused to either mouse ERα (as a positive control) or mcER-like ligand binding domain (LBD)) or pSG5-Gal4-mcERR LBD were cloned into pSG5-VP16 to create VP16-GAL4-ER and VP16-GAL4-mcERR expression vectors (Paulmurugan et al., 2009). For ligand screening assays HEK-293 cells were first seeded into T150 flasks containing charcoal-stripped Dulbecco's Modified Eagle Medium (DMEM; Sigma–Aldrich, UK) and confluent cells were used to prepare a cell suspension (30% confluence). One hundred μL of the cell suspension was then added to each well of a 96 well plate and incubated overnight. Cells were then transfected with 15 ng pGL3-GAL4 firefly luciferase reporter construct and 10 ng pRL-TK renilla luciferase (to normalise transcriptional responses for transfection efficiencies) using Fugene 6 (Roche). Mock wells (no chemical and no ER) were transfected with 30 ng of empty pSG5, whereas treatment wells were transfected with 20 ng pSG5 and 10 ng of the appropriate VP16-GAL4 expression plasmid containing either mouse or mollusc ER or mollusc ERR LBD. Twenty four hours post transfection, cells were washed 2–3 times with 100 μL of fresh medium per well and cells were incubated with 100 μL media containing test compounds (E2, EE2, BPA, 4-*t*-OP, Tam-OH, ICI 182,780, CPA, DES, GEN or MT) in duplicate at concentrations ranging from 10^−4^ M to 10^−10^ M, or with media containing solvent (ethanol) without chemical (final concentration of ethanol 0.01% per well in all cases). After 24 h cells were lysed by removing 50 μL of cell culture medium and replacing with 50 μL of 2× LucLite™ reagent (Packard; prepared according to the manufacturer's instructions). Cells were incubated in the dark for 15 min until lysis was complete. Reactions were transferred to a white 96-well microtitre plate and luminescence was measured on a Wallac Victor2 1420 luminometer. Renlight reagent was prepared by diluting Coelenterazine substrate (stock 1 mg/mL DMSO) 100-fold in Renilla buffer (0.5 M HEPES, pH 7.8, 40 mM EDTA). Twenty five μL of Renlight reagent was then added to each well, allowed to equilibrate for 30 min at room temperature, and luminescence readings were taken once again. Firefly luciferase readings were then normalised using renilla luciferase values to account for experimental variation.

### 2.8 Statistical analysis of gene transcription

Statistical analysis of gene transcription was undertaken by Dr. Nelly van der Hoeven (Ecostat, The Netherlands). To determine whether exposure to 4-*t*-OP or E2 affected gene transcription in any of the tissues at any of the time points, the following model (Formula 1) was fitted to the QPCR data:

$$\text{Formula}\;1:\;Y_{ijkl}=\mu +\tau_{i}+S_{j}+{\left(\tau s\right)}_{ij}+e_{ijk}+d_{ijkl}$$

where *μ* is the overall mean value, *τ*_*i*_ the effect of the *i*-th treatment level, *s*_*j*_ the effect of the *j*-th sex, (*τs*)_*ij*_ the effect of the interaction between treatment and sex, *e*_*ijk*_ error term describing the (normal distributed) error between vessels and *d*_*ijkl*_ the error term describing the (normal distributed) error between individuals within vessels. In analysing the data with this model, the error within the vessels and between the vessels is distinguished. The analysis was performed in S-plus 2000 using the expression aov(formula = response∼ Sex + Treatment + Treatment:Sex + Error(Treat.Vessel), data = data) for the analysis of the treatment effect and aov(formula = response∼ Treatment + Sex + Treatment:Sex + Error(Treat.Vessel), data = data) for the analysis of the effect of the sex. Here ‘aov’ is analysis of variance (used to analyse data with a numeric response and one or more discrete regression variables), ‘response∼’ represents the ER or ERR gene transcriptional response in the tissue under consideration, ‘Sex + Treatment’ are the main experimental variables and ‘Treatment:Sex’ are the two variables as an interaction term in the model.

For the factor “sex” and the interaction term, *p*-values were assessed both within and between the test vessels. These two *p*-values were considered as independent observations on the same variable. They were combined using the equation shown in Formula 2:

$$\text{Formula}\;2:\;T=-2x[\log(p_{\text{within}})+\log(p_{\text{between}})]$$

where under the null-hypothesis that the factor or interaction term had no effect, *T* is chi-square distributed with 4 degrees of freedom (Hedges and Olkin, 1985).

Although the experiment was not designed to test for the effects of treatment on mollusc reproduction, the correlation between egg production and the qPCR data at any of the time points was investigated with nine ANOVAs performed using the egg production per female as the dependent variable and the female vessel mean of each of the other end-points as predicting variable. To correct for potential influence of the treatment, the treatment was used as second factor in these ANOVAs.

## 3 Results

### 3.1 *In vivo* exposure: chemical dosing

Analysis of biological activity of water extracts dosed with E2 using the YES assay found concentrations of E2 to be close to nominal values (Table 1). The limit of detection for E2 in tank water (based on C18 solid phase extraction of a 1 litre volume) using the YES was 0.1 ng/L. No estrogenic activity was detected in either the distilled water control or solvent control (SC) at any time point measured. Biological activity of the water in the treated tanks increased in a concentration-dependent manner in the treatments from 8.41 ± 3.92 ng/L to 152.69 ± 69 and 1009.33 ± 154.93 ng/L (mean ± SD of each individual replicate exposure concentration at each time point) for 10, 100 and 1000 ng E2/L nominal exposure concentrations, respectively. There was no 4-*t*-OP detected in the SC at any time point measured. The mean concentration of 4-*t*-OP was 5.6 ± 0.62 μg/L and 28.24 ± 8.99 μg/L (mean ± SD of each individual replicate exposure concentration at each time point) for 5 μg 4-*t*-OP/l and 25 μg 4-*t*-OP/l nominal concentration, respectively.

### 3.2 *In vivo* exposure: mRNA expression of the studied genes

Table 2 shows *p*-values for the hypothesis that the factor “treatment”, the factor “sex” and the interaction between these two factors does not affect the toxicological end-point given after 1, 6 and 12 weeks exposure. The mean and standard deviations of all observed values of mRNA expression of the studied genes in each tissue of each snail for both sexes throughout the exposure are presented in Tables S1 (week 1), S2 (week 6) and S3 (week 12).

**Fig. 1:**
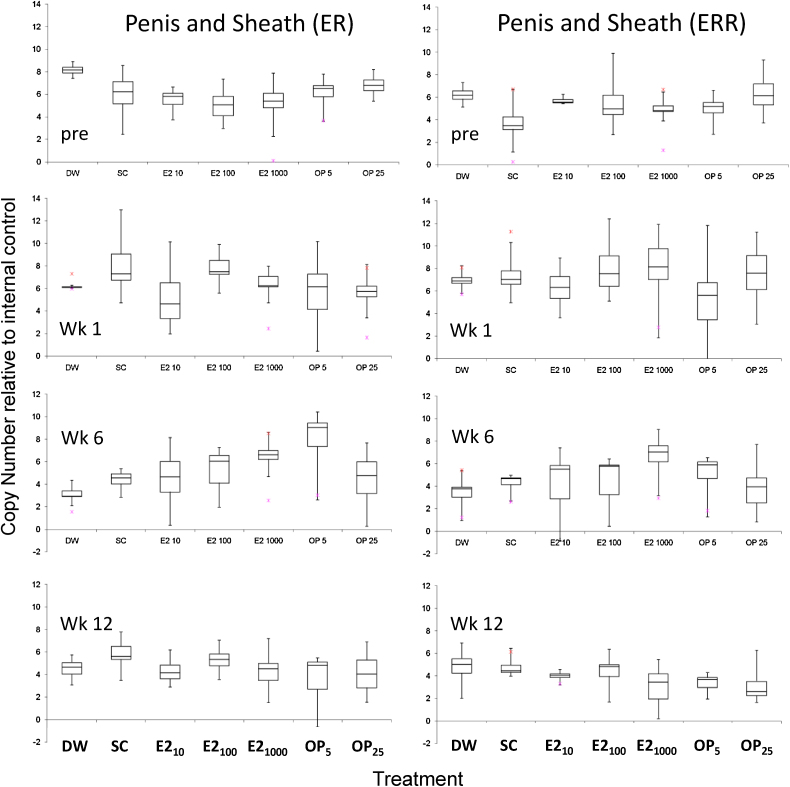
Bar and Whisker plots showing the mRNA expression levels of mcER-like and mcERR genes in the penis and sheath assessed by aQPCR at pre-exposure (pre), and after 1 week (wk1). 6 week (wk6), and 12 week (wk12) exposure to 17β-estradiol (E2; 10, 100 and 1000 ng/L), 4-*tert*-Octylphenol (OP; 5 and 25 μg/L) or the water (DW) and solvent controls (SC). Week 1 DW *n* = 5♂; SC *n* = 5♂; E2 10 *n* = 4♂, E2 100 *n* = 5♂; E2 1000 *n* = 5♂; OP 5 *n* = 8♂; OP 25 *n* = 6♂. Week 6 DW *n* = 5♂; SC *n* = 4♂; E2 10 *n* = 7 ♂, E2 100 *n* = 5 ♂; E2 1000 *n* = 6♂; OP 5 *n* = 4♂; OP 25 *n* = 6♂; Week 12 DW *n* = 6♂; SC *n* = 5♂; E2 10 *n* = 4♂, E2 100 *n* = 5♂; E2 1000 *n* = 6♂; OP 5 *n* = 6♂; OP 25 *n* = 4♂ The ends of the whisker are set at 1.5*IQR above the third quartile (Q3) and 1.5*IQR below the first quartile (Q1). If the Minimum or Maximum values are outside this range, then they are shown as outliers (*). Equivalent plots of the responses of mcER-like and mcERR genes in male and female ganglia, gonad-digestive complex and the albumin gland (female only) can be found in Supplementary information Figures S2–S6).

Sexual dimorphism occurs for the mRNA expression of mcERR and mcER-like genes in the reproductive organs (penis sheath and gonad-digestive complex) and levels are statistically significantly higher (*p* < 0.05) in males than in females. In contrast, no significant difference in mcER-like and mcERR expression between males and females was found in the cerebral ganglia (Table 2). Significant interactive effects (effects of treatment on mRNA expression of the studied genes related to snail sex) occurred in reproductive tissues (albumen gland and penis and penis sheath) at week 1 and week 6 (Table 2). At week 1, mean mRNA expression of mcER-like gene in the albumen gland of the females (S2) almost doubled in the 10 ng E2/L treatment (1.54 ± 1.43) relative to the solvent control (0.88 ± 1.03). At week 6, mean mRNA expression levels of mcER-like gene in the penis and penis sheath of males in the 5 μg/L treatment (7.79 ± 3.21) were greater than those of the solvent control (4.41 ± 0.76) (Fig. 1). However, no significant differences in mean gene transcription levels relative to the solvent control could be discerned in any treatment group due to the variability between individuals both within and between treatments. The solvent control was not significantly different from the water control at any time point measured (*p* > 0.05).

### 3.3 *In vivo* exposure: relationship between egg production and the other end-points

No relationships between egg production per female and mcER-like and mcERR expression was observed (Table S4). A weak (but statistically significant; *p* = 0.040) negative correlation between female wet-weight and egg production was found (Table S4). After correcting for the treatment level, the mean egg production in this experiment decreased by about 10 eggs per 0.1 g increase in snail weight. However, it was not possible to discern the reproductive effort (and therefore contribution) that individual females made to the number of egg masses in each tank. There was no significant effect of treatment on mean cumulative egg production per female (Figure S7) over the 12 week exposure period.

### 3.4 Gel mobility shift assays

Electrophoretic mobility shift assays (EMSAs) showed that the recombinant mcER-like DNA binding domain was able to specifically recognise and bind to the core consensus estrogen response element (ERE) inverted palindromic DNA sequence (5′-GGTCAnnnTGACC-3′) in the absence of a ligand (Figure S1). Mouse ERα was used as a positive control and showed binding to the ERE which could be displaced using unlabelled competitor ERE.

### 3.5 Cell transfection assays

**Fig. 2:**
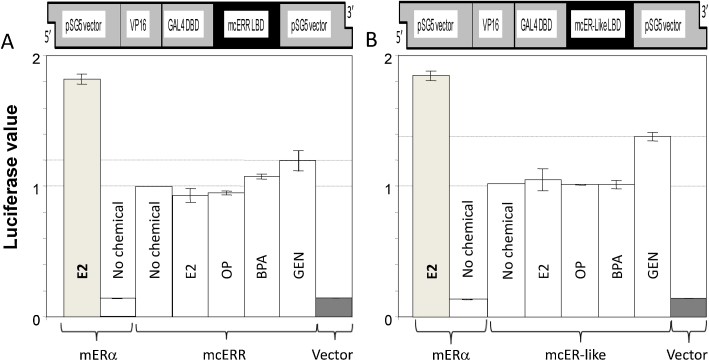
Response of the VP16-mcERR(LBD)-Gal4 (A) and VP16-mcER-like(LBD)-Gal4 (B) construct transfected in HEK293 cells in the presence and absence of a chemicals (one dose from a single assay is presented). A recombinant plasmid was constructed by restriction cloning using the mcER-like or mcERR LBD, a GAL4 DNA binding domain and a VP16 transactivation domain in pSG5 expression vector (Stratagene) shown above the charts. This construct was co-transfected into HEK293 cells along with a luciferase reporter coupled to a GAL4 DNA response element and a renilla plasmid used to control for transfection efficiency. Following transfection, cells were treated for 24 h with a suite of chemicals at a range of concentrations from to 100 pM to 100 μM. Luciferase and renilla activites were measured by luminometer. Response at 100 nm is shown for 17β-estradiol (E2), Response at 1 μM is shown for all other chemicals. OP, 4-*tert*-Octylphenol; BPA, bisphenol A; GEN, genistein. Luciferase counts were normalised to renilla counts to control for transfection efficiency. The data presented here was normalised to the constitutive activity observed when the mcERR (A) or mcER-like (B) construct was transfected and left untreated. A construct containing mouse ERα LBD was used as a positive control. A construct with the LBD excised (vector) was used as a negative control. Error bars represent one standard deviation of the mean from replicate wells.

**Fig. 3:**
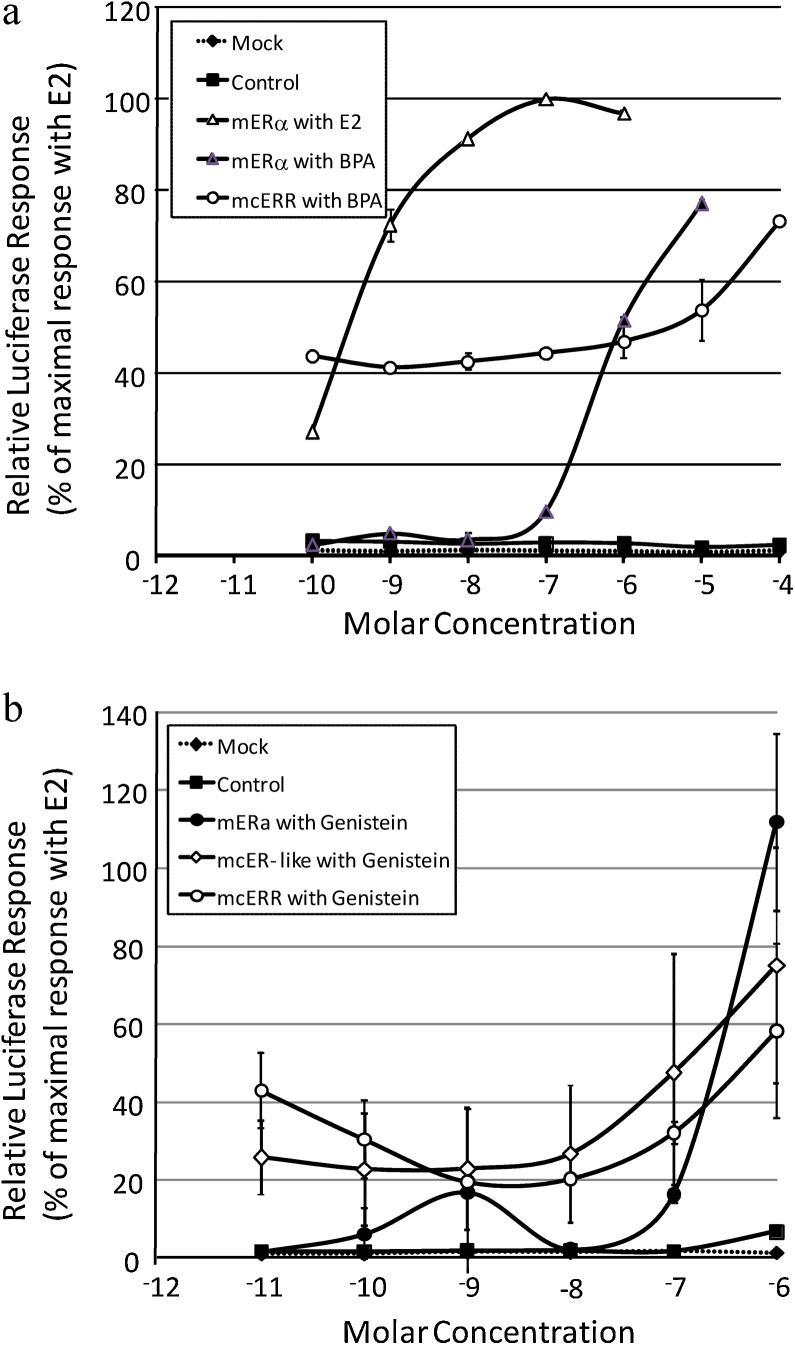
Examples of dose responses of transfected HEK293 cells containing mouse ER (mERα), marisa ER-like (mcER) or marisa ERR (mcERR) exposed to various chemicals. A) Response of mERα to E2 (10^−10^ to 10^−6^ M) and BPA (10^−10^ to 10^−5^ M) and mcERR to BPA (10^−10^ to 10^−4^ M). B) Response of mERα, mcER and mcERR to genistein from 10^−11^ to 10^−4^ M. The inclusion of the construct with the LBD excised plus chemical (Mock) and mERα with solvent carrier (control) were used as negative controls. Error bars denote the standard deviation of two replicate samples per treatment. Concentrations above 10^−6^ M showed signs of toxicity and are therefore not included.

The response of mERα, mcER-like and mcERR to a single concentration of E2, 4-*t*-OP, GEN and BPA using the transfected HEK293 cell assay is shown in Fig. 2. Both mcER-like and mcERR LBDs conferred constitutive activation of the luciferase reporter at a level between 30 and 50% of that observed with mouse ERα LBD treated with 100 nM E2 (positive ‘maximal activation’ control). Activation of the luciferase reporter by mouse ERα LBD in the absence of ligand was between 1 and 10% of the maximum response. Treatment of the mcER-like-transfected cells with the known vertebrate ER agonists E2 or 4-*t*-OP (also tested *in vivo*) had no effect on the constitutive activity of the luciferase reporter. Moreover, with the exception of GEN (and possibly BPA at the higher concentrations tested), various known agonists (EE2 and DES), antagonists (Tam-OH, ICI 182,780), androgens (MT) and anti-androgens (CPA) had no effect on mean baseline transcriptional responses either. GEN was able to increase the transcriptional response of both mollusc receptors consistently in repeated assays, and in one assay, increased basal transcription of both mcERR and mcER-like by 3- and 6-fold, respectively, at 10^−6^ M (see Fig. 3B). In contrast, GEN elevated the transcriptional response of the mouse ERα from <10% to a supramaximal response (115%) at concentrations between 10^−9^ and 10^−6^ M. BPA consistently increased transcription of the mcERR between 1.1-fold (at 10^−6^ M) and up to 2-fold (at 10^−4^ M) in repeated assays, resulting in a shallow dose-response curve between 10^−8^ and 10^−4^ M (Fig. 3A). There was no effect of BPA on mcER-like in repeated tests. In contrast BPA increased mean transactivation of mERα from <10 to 100% at concentrations between 10^−8^ M and 10^−5^ M.

## 4 Discussion

In this paper we describe a range of experiments to investigate whether protein activation *in vitro* and gene transcription *in vivo* of ER-like and/or ERR in the gastropod snail *M. cornuarietis* (Bannister et al., 2007) are affected by exposure to a range of known vertebrate endocrine disrupting chemicals. Using previously published results (Oehlmann et al., 2000) as our stimulus, we set out to establish whether the main vertebrate steroid estrogen (E2) or the estrogenic alkylphenol (4-*t*-OP) could affect mRNA expression of mcER-like and/or mcERR in *M. cornuarietis*, in an *in vivo* exposure experiment. Exposure levels were not necessarily representative of environmental levels, as the purpose was to explore whether estrogenic chemicals affected transcription in various tissues of the snails. Patterns of gene transcription in the absence of chemical were consistent with previous findings (Bannister et al., 2007) and showed clear tissue and sex-specific patterns, with the highest levels of mRNA expression of both mcER-like and mcERR gene in the male reproductive organs (penis and penis sheath). There was no significant effect of treatment (with 95% confidence) on the mRNA expression of mcER-like and mcERR genes *in vivo* after 1, 6 and 12 weeks exposure. However, we cannot eliminate the possibility that much more rapid (early) responses resulting from exposure to the chemicals may occur. Indeed, early effects (1 day post treatment) on mollusc ER transcription following exposure to EE2 and BPA have been suggested in *Potamopyrgus antipodarum* (Stange et al., 2011), although only subtle changes (∼half to 2-fold increase) in gene transcription in pooled individuals exposed to a single nominal dose were reported. Statistical analysis of our data suggests that changes in gene expression are more likely to occur in reproductive tissues. Using our sample size of ∼6 snails/treatment/timepoint we found an 84% and 90% chance of an effect of treatment on mRNA expression of mcER-like gene in the reproductive organs and cerebral ganglia, respectively, in week 6 and an 88% chance of an effect of treatment on mRNA expression of mcERR gene in the reproductive tract in week 1. Although not statistically significant (with 95% confidence), these odds contrast with the much lower likelihood (in the order of 30%) of an effect of treatment on mRNA expression of mcER-like and mcERR genes in the gonad-digestive complex or on mRNA expression of mcERR gene in the cerebral ganglia at all time points measured. Whether these indicators are (i) simply a consequence of inter-individual variability within the various treatment groups or (ii) real effects of treatment manifest in a few individuals, is presently unknown. However, given the fact that E2 and 4-*t*-OP were inactive *in vitro* on the LBD of mcER-like suggests that the former explanation would be most likely.

Transcriptional changes, however, reflect only one facet of nuclear receptor gene expression, and consequently of their physiological control over homeostasis, development, metabolism and reproduction. Indeed, nuclear receptor function is also known to be regulated by post-translational modifications, including methylation, phosphorylation, sulfonation and acetylation (of specific amino acid residues serine, tyrosine, threonine and lysine) or addition of other proteins or polypeptides (*e.g.* sumoylation and ubiquitination). Such modifications are known to have profound influences on the clinical outcome of endocrine related diseases (Anbalagan et al., 2012). Post-translational modification (O-sulfonation) to serine and threonine residues of cytoskeletal proteins, proteases and orphan receptors is reported in the mollusc *Lymnaea stagnalis*, the unicellular parasite *Plasmodium falciparum* and in humans, highlighting the importance of physiological control beyond transcriptional changes across eukaryotes generally (Medzihradszky et al., 2004). Furthermore, modulation of transcriptional and translational control by miRNAs (a vital and evolutionarily ancient component of genetic regulation) adds a further level of complexity (Lee et al., 2007) which has now also been shown to be subject to control by chemicals, including endocrine disruptors (Tilghman et al., 2012). The effects of E2 and 4-*t*-OP on post-translational and/or miRNA control of nuclear receptor translation and function were not investigated in this study, and cannot therefore be discounted.

We found no association between mRNA expression of mcER-Like or mcERR genes, treatment and egg production in *M. cornuarietis*. Although this analysis contributes to the debate on the effects of chemicals on mollusc reproduction, the large variability in egg mass production between replicate tanks, the short exposure duration and the need to sub-sample snails (for effects of treatment on transcription) throughout the exposure precludes a robust analysis and interpretation of the effects of treatment on egg production. Also, as reproductive output could not be measured in individual snails within the treatment groups, it was not possible to investigate whether egg production was associated with gene expression using these data.

An important determinant of gene promoter selectivity of activated ERs in vertebrates is their ability to bind to sequences of DNA called estrogen response elements (EREs) within the promoter of target genes (Hall et al., 2001). Using gel-shift assays we found that mcER-like can bind to consensus EREs, like mouse ERα does. Our present ignorance about the presence, position and extent of EREs within the genome of molluscs, however, means that the functional significance of this finding is presently unknown. In vertebrates, the ERR and ERα have both been found to bind the classical ERE (Vanacker et al., 1999). The apparent absence of binding of mcERR to the ERE (data not shown) may suggest less functional overlap between ER and ERR orthologues in molluscs than in vertebrates.

Similar to reports in other molluscs (Thornton et al., 2003), mcER-like and mcERR constitutively activated reporter gene expression in the absence of E2 to levels around 40% of the maximal response of mouse ER in the presence of E2. Moreover, consistent with the *in vivo* results, neither the steroid estrogen E2, the xenoestrogen 4-*t*-OP nor a range of other potential ligands had any obvious consistent dose-response effect on the constitutive activity of mcER-like and mcERR. Tentatively positive *in vitro* results were, however, obtained with the phytoestrogen genistein (mcER-like) and the xenoestrogen bisphenol-A (mcERR). The fact that genistein increased basal activity of reporter gene expression with mcER-like (and to a lesser extent mcERR) LBDs in our system suggests a possible modulatory role of some phytoestrogens on the mollusc ER (and possibly ERRs), similar to those reported in vertebrates (Suetsugi et al., 2003; Kuiper et al., 1998; Mueller et al., 2004) and more recently in an ascidian urochordate (Park et al., 2009). Moreover, the weak interaction between the mcERR LBD and BPA in our study is consistent with a previous report of an interaction between BPA and the human ERRγ (Takayanagi et al., 2006).

## 5 Conclusion

These data provide no evidence that *M. cornuarietis* reproduction is highly susceptible to E2 or 4-*t*-OP over 12 weeks exposure, or that the mollusc ER and ERR gene transcription in various tissues are responsive to a vertebrate steroid estrogen. The discovery of a weak interaction of genistein and BPA with mcER-like and mcERR LBDs, respectively, *in vitro* suggests that ligands (both natural and anthropogenic) for these receptors may exist which could provide important insights into the functional role of mollusc ERs and ERRs *in vivo*. Finally, given the huge diversity of gastropod molluscs, the generality of these findings to other species of mollusc needs to be established.

## Conflict of interest

The authors have nothing to disclose.

## Figures and Tables

**Table 1:** Measurements (mean ± SD) of 17β-estradiol (E2) and 4-t-Octylphenol (OP) determined in the water from the *in vivo* exposure experiment at different time points and in triplicate tanks. E2 measurements were calculated based on activity of extracts of water in the YES assay, whereas OP measurements were determined by HPLC. DW is distilled water control, SC is the solvent control. ND, not detected.

	OP (μg/L)			E2 (ng/L)				
	SC	5	25	DWC	SC	10	100	1000
Week 3	ND	6.3 ± 0.4	28.9 ± 1.1	ND	ND	6.4 ± 0.5	84.7 ± 49.5	878 ± 234
Week 6	ND	5.9 ± 0.4	22.3 ± 0.8	ND	ND	23.3 ± 6.3	145.3 ± 24.4	1070 ± 45.8
Week 9	ND	5.4 ± 0.3	41.7 ± 4.3	ND	ND	7.4 ± 0.7	228.0 ± 21.0	1080 ± 17.3
Week 10	ND	4.9 ± 0.3	20.1 ± 0.3					

**Table 2:** The *p*-value for the test of the main factors sex and treatment (*T*) level and the interaction (*i*) between these two. For the factor “sex” and the interaction term, *p*-values were calculated for both within and between the test vessels. These two *p*-values were combined.

Toxicological end-point	*p*-Values week 1			*p*-Values week 6			*p*-Values week 12		
	*T*	Sex	*i*	*T*	Sex	*i*	*T*	Sex	*i*
ER-like (cerebral ganglia)	0.31	0.17	0.39	0.10	0.72	0.08	0.75	0.77	0.39
ER-like in penis and sheath (M) or albumen gland (F)	0.74	<10^−5^	0.010	0.16	<10^−5^	0.006	0.74	<10^−5^	0.74
ER-like (gonad-digestive complex)	0.65	2 × 10^−5^	0.79	0.59	0.001	0.92	0.86	1 × 10^−5^	0.56
ERR (cerebral ganglia)	0.90	0.053	0.27	0.51	0.63	0.58	0.79	0.44	0.93
ERR penis and sheath (M) or albumen gland (F)	0.12	<10^−5^	0.11	0.44	<10^−5^	0.33	0.45	<10^−5^	0.51
ERR (gonad-digestive complex)	0.62	<10^−5^	0.81	0.60	<10^−5^	0.65	0.60	<10^−5^	0.23
Wet weight	0.11	0.003	0.020	0.89	<10^−5^	0.60	0.32	<10^−5^	0.34
Shell height	0.15	0.082	0.061	0.88	2 × 10^−5^	0.69	0.29	<10^−5^	0.63
Aperture width	0.30	0.34	0.10	0.84	4 × 10^−4^	0.67	0.46	2 × 10^−5^	0.75
