# Supplementary material for: No substantial changes in estrogen receptor and estrogen-related receptor orthologue gene transcription in Marisa cornuarietis exposed to estrogenic chemicals
Source: Aquat Toxicol. 2013 Sep 15;140-141:19–26. doi: 10.1016/j.aquatox.2013.05.002 (PMC3778743; doi:10.1016/j.aquatox.2013.05.002)
Supplement: Supplementary file 1 [file mmc1.docx]

**Supplementary Information**

Figure S1. DNA binding properties of mcER-like and mouse ER were investigated using electrophoretic mobility shift assays (EMSAs). The mcER-like coding region was cloned into pSG5 expression vector (Stratagene) and recombinant protein was produced using the TNT T7 Quick-Coupled Transcription/Translation system (Promega). The sizes of recombinant proteins synthesised were assessed by incorporation of ^35^S methionine into transcription/translation reactions run in parallel. Hot proteins were resolved by polyacrylamide gel electrophoresis and detected by autoradiography. Unlabelled recombinant protein was incubated in the presence of radiolabelled double stranded DNA encoding a consensus oestrogen response element (ERE). Reactions were incubated in the presence of either non-radiolabelled specific DNA competitor, or non-specific DNA competitor to assess the specificity of binding occurring to radiolabelled DNA. DNA and protein were resolved on a TBE/polyacylamide gel, which was subjected to subsequent drying and autoradiography. mcER-like specifically bound the consensus ERE, as did the positive control (mouse (m)ERα).

| toxicological end-point |  |  | Dilution water | Solvent control | 17b-estradiol, 10 ng/l | 17b-estradiol, 100 ng/l | 17b-estradiol, 1000 ng/l | octyl-phenol , 5 μg/l | octyl-phenol , 25 μg/l |
| --- | --- | --- | --- | --- | --- | --- | --- | --- | --- |
| ER-like product in cerebral ganglia | mean | female | 2.95 | 2.79 | 3.40 | 3.70 | 3.03 | 2.76 | 2.58 |
|  |  | male | 2.49 | 2.59 | 3.55 | 4.45 | 2.65 | 3.76 | 3.43 |
|  | SD | female | 0.65 | 0.56 | 1.11 | 0.82 | 1.02 | 1.09 | 0.67 |
|  |  | male | 1.51 | 1.29 | 1.80 | 1.53 | 0.75 | 1.26 | 0.59 |
|  |  |  |  |  |  |  |  |  |  |
| ER-like product in penis and sheath (M) or albumen gland (F) | mean | female | 0.94 | 0.88 | 1.54 | 0.92 | 1.40 | 1.09 | 1.39 |
|  |  | male | 6.35 | 7.95 | 5.22 | 7.62 | 5.83 | 5.94 | 5.42 |
|  | SD | female | 0.77 | 1.03 | 1.43 | 0.43 | 1.59 | 0.16 | 0.97 |
|  |  | male | 0.55 | 2.30 | 2.38 | 1.20 | 1.94 | 2.39 | 2.06 |
|  |  |  |  |  |  |  |  |  |  |
| ER-like product in gonad-digestive complex | mean | female | 0.90 | 0.99 | 1.02 | 0.99 | 0.95 | 1.07 | 1.26 |
|  |  | male | 1.28 | 1.42 | 1.37 | 1.40 | 1.12 | 1.59 | 1.79 |
|  | SD | female | 0.49 | 0.26 | 0.45 | 0.26 | 0.25 | 0.30 | 0.51 |
|  |  | male | 0.20 | 0.30 | 0.24 | 0.29 | 0.32 | 0.53 | 0.58 |
|  |  |  |  |  |  |  |  |  |  |
| ERR product in cerebral ganglia | mean | female | 2.77 | 2.62 | 2.50 | 2.22 | 2.74 | 2.92 | 2.79 |
|  |  | male | 1.66 | 2.76 | 2.83 | 2.64 | 2.63 | 3.15 | 2.48 |
|  | SD | female | 0.71 | 0.67 | 0.67 | 0.46 | 1.24 | 0.55 | 1.29 |
|  |  | male | 0.94 | 1.50 | 1.21 | 1.08 | 0.76 | 1.04 | 0.61 |
|  |  |  |  |  |  |  |  |  |  |
| ERR product in penis and sheath (M) or albumen gland (F) | mean | female | 0.93 | 0.72 | 1.48 | 0.93 | 1.26 | 0.69 | 1.47 |
|  |  | male | 6.93 | 7.63 | 6.33 | 8.05 | 7.61 | 5.58 | 7.45 |
|  | SD | female | 0.83 | 1.02 | 1.44 | 0.55 | 1.39 | 0.17 | 0.96 |
|  |  | male | 0.86 | 2.22 | 1.50 | 1.94 | 2.99 | 2.76 | 2.05 |
|  |  |  |  |  |  |  |  |  |  |
| ERR product in gonad-digestive complex | mean | female | 0.72 | 0.71 | 0.89 | 0.62 | 0.91 | 0.72 | 0.84 |
|  |  | male | 1.40 | 1.49 | 1.46 | 1.35 | 1.61 | 1.48 | 1.61 |
|  | SD | female | 0.31 | 0.28 | 0.25 | 0.11 | 0.29 | 0.04 | 0.28 |
|  |  | male | 0.39 | 0.83 | 0.27 | 0.40 | 0.43 | 0.63 | 0.45 |
|  |  |  |  |  |  |  |  |  |  |
| Wet.weight  (g) | mean | female | 4.37 | 5.65 | 4.06 | 5.15 | 4.26 | 4.89 | 3.71 |
|  |  | male | 3.49 | 3.66 | 4.22 | 3.94 | 3.78 | 3.70 | 4.17 |
|  | SD | female | 1.02 | 1.87 | 1.39 | 1.46 | 1.45 | 0.83 | 1.01 |
|  |  | male | 1.02 | 0.53 | 0.63 | 0.78 | 0.89 | 0.56 | 1.58 |
|  |  |  |  |  |  |  |  |  |  |
| Shell.height  (mm) | mean | female | 27.08 | 29.51 | 26.29 | 28.67 | 26.41 | 27.93 | 25.32 |
|  |  | male | 25.22 | 25.92 | 26.98 | 26.74 | 26.04 | 26.06 | 27.40 |
|  | SD | female | 2.62 | 3.39 | 3.42 | 2.84 | 2.78 | 1.64 | 2.55 |
|  |  | male | 3.01 | 2.49 | 0.94 | 1.83 | 2.25 | 2.02 | 3.43 |
|  |  |  |  |  |  |  |  |  |  |
| Aperture.width  (mm) | mean | female | 14.07 | 14.86 | 13.48 | 14.41 | 13.26 | 14.08 | 12.85 |
|  |  | male | 12.74 | 13.30 | 14.13 | 13.82 | 13.44 | 13.29 | 14.30 |
|  | SD | female | 0.84 | 2.03 | 1.64 | 1.33 | 1.25 | 1.23 | 1.52 |
|  |  | male | 1.58 | 0.87 | 0.42 | 0.93 | 0.90 | 0.98 | 1.69 |
|  |  |  |  |  |  |  |  |  |  |
| number of snails |  | female | 6 | 7 | 8 | 7 | 7 | 4 | 6 |
|  |  | male | 5 | 5 | 4 | 5 | 5 | 8 | 6 |

Table S1. The mean and standard deviation of the gene expression of two genes in three tissues in females and males and the size of the snails after 1 week exposure.

| toxicological end-point |  |  | Dilution water | Solvent control | 17b-estradiol, 10 ng/l | 17b-estradiol, 100 ng/l | 17b-estradiol, 1000 ng/l | octyl-phenol , 5 μg/l | octyl-phenol , 25 μg/l |
| --- | --- | --- | --- | --- | --- | --- | --- | --- | --- |
| ER-like product in cerebral ganglia | mean | female | 2.63 | 2.97 | 2.80 | 2.52 | 1.92 | 2.57 | 2.92 |
|  |  | male | 2.97 | 2.94 | 3.28 | 2.08 | 2.54 | 2.66 | 2.08 |
|  | SD | female | 0.57 | 1.10 | 0.81 | 0.45 | 0.47 | 1.70 | 0.31 |
|  |  | male | 0.24 | 0.84 | 0.77 | 0.27 | 0.36 | 0.33 | 0.48 |
|  |  |  |  |  |  |  |  |  |  |
| ER-like product in penis and sheath (M) or albumen gland (F) | mean | female | 0.50 | 0.73 | 0.81 | 0.88 | 0.88 | 0.76 | 0.71 |
|  |  | male | 2.95 | 4.41 | 4.57 | 5.50 | 6.26 | 7.79 | 4.50 |
|  | SD | female | 0.32 | 0.29 | 0.26 | 0.30 | 0.17 | 0.64 | 0.18 |
|  |  | male | 0.87 | 0.76 | 1.85 | 1.38 | 1.99 | 3.21 | 1.86 |
|  |  |  |  |  |  |  |  |  |  |
| ER-like product in gonad-digestive complex | mean | female | 0.79 | 1.10 | 0.83 | 0.78 | 0.85 | 1.10 | 0.95 |
|  |  | male | 1.27 | 1.40 | 1.15 | 1.03 | 0.91 | 1.39 | 1.18 |
|  | SD | female | 0.32 | 0.39 | 0.31 | 0.14 | 0.32 | 0.47 | 0.16 |
|  |  | male | 0.28 | 0.24 | 0.45 | 0.40 | 0.45 | 0.28 | 0.43 |
|  |  |  |  |  |  |  |  |  |  |
| ERR product in cerebral ganglia | mean | female | 1.44 | 2.03 | 1.95 | 1.67 | 1.97 | 1.60 | 1.48 |
|  |  | male | 1.77 | 1.55 | 1.82 | 1.54 | 2.46 | 2.07 | 1.39 |
|  | SD | female | 0.56 | 0.72 | 0.84 | 0.53 | 0.59 | 0.80 | 0.25 |
|  |  | male | 0.40 | 0.32 | 0.45 | 0.43 | 0.56 | 0.31 | 0.31 |
|  |  |  |  |  |  |  |  |  |  |
| ERR product in penis and sheath (M) or albumen gland (F) | mean | female | 0.58 | 0.82 | 0.62 | 0.96 | 0.83 | 0.48 | 0.45 |
|  |  | male | 3.49 | 4.24 | 4.57 | 4.86 | 6.53 | 4.99 | 4.03 |
|  | SD | female | 0.44 | 0.60 | 0.29 | 0.50 | 0.36 | 0.32 | 0.04 |
|  |  | male | 1.53 | 1.09 | 2.03 | 1.60 | 1.95 | 2.12 | 1.79 |
|  |  |  |  |  |  |  |  |  |  |
| ERR product in gonad-digestive complex | mean | female | 0.42 | 0.49 | 0.54 | 0.43 | 0.58 | 0.51 | 0.43 |
|  |  | male | 1.22 | 0.96 | 1.45 | 1.05 | 0.94 | 1.34 | 1.22 |
|  | SD | female | 0.20 | 0.17 | 0.12 | 0.11 | 0.23 | 0.17 | 0.10 |
|  |  | male | 0.32 | 0.28 | 1.02 | 0.44 | 0.39 | 0.29 | 0.87 |
|  |  |  |  |  |  |  |  |  |  |
| Wet.weight  (g) | mean | female | 5.68 | 5.56 | 5.93 | 5.10 | 5.59 | 5.20 | 5.46 |
|  |  | male | 3.98 | 4.27 | 4.12 | 4.21 | 4.72 | 4.46 | 4.21 |
|  | SD | female | 0.67 | 0.72 | 0.61 | 0.88 | 0.78 | 1.10 | 1.10 |
|  |  | male | 0.92 | 0.59 | 0.79 | 1.01 | 1.05 | 0.32 | 1.08 |
|  |  |  |  |  |  |  |  |  |  |
| Shell.height  (mm) | mean | female | 30.18 | 29.57 | 30.01 | 28.61 | 29.13 | 28.59 | 29.46 |
|  |  | male | 26.42 | 27.20 | 26.87 | 26.81 | 28.31 | 27.66 | 26.81 |
|  | SD | female | 1.25 | 1.31 | 0.89 | 1.66 | 1.40 | 2.15 | 1.75 |
|  |  | male | 2.06 | 1.13 | 2.07 | 2.15 | 2.85 | 0.88 | 2.66 |
|  |  |  |  |  |  |  |  |  |  |
| Aperture.width  (mm) | mean | female | 14.80 | 14.72 | 15.13 | 14.43 | 14.48 | 14.26 | 14.96 |
|  |  | male | 13.58 | 13.85 | 13.81 | 13.53 | 14.56 | 13.99 | 13.55 |
|  | SD | female | 1.08 | 0.63 | 0.58 | 0.72 | 0.46 | 1.08 | 0.82 |
|  |  | male | 0.97 | 0.58 | 0.89 | 0.85 | 1.06 | 0.39 | 1.17 |
|  |  |  |  |  |  |  |  |  |  |
| number of snails |  | female | 7 | 8 | 5 | 7 | 6 | 7 | 6 |
|  |  | male | 5 | 4 | 7 | 5 | 6 | 4 | 6 |

Table S2. The mean and standard deviation of the gene expression of two genes in three tissues in females and males and the size of the snails after 6 weeks exposure.

| toxicological end-point |  |  | Dilution water | Solvent control | 17b-estradiol, 10 ng/l | 17b-estradiol, 100 ng/l | 17b-estradiol, 1000 ng/l | octyl-phenol , 5 μg/l | octyl-phenol , 25 μg/l |
| --- | --- | --- | --- | --- | --- | --- | --- | --- | --- |
| ER-like product in cerebral ganglia | mean | female | 1.73 | 2.44 | 2.58 | 2.07 | 2.13 | 2.01 | 2.39 |
|  |  | male | 1.97 | 2.65 | 1.95 | 2.25 | 2.46 | 2.49 | 1.63 |
|  | SD | female | 0.37 | 0.39 | 0.75 | 0.39 | 0.49 | 0.87 | 0.80 |
|  |  | male | 1.21 | 0.75 | 0.67 | 0.50 | 0.68 | 0.86 | 0.36 |
|  |  |  |  |  |  |  |  |  |  |
| ER-like product in penis and sheath (M) or albumen gland (F) | mean | female | 0.76 | 0.73 | 0.81 | 0.96 | 0.81 | 0.67 | 0.83 |
|  |  | male | 4.58 | 5.63 | 4.33 | 5.35 | 4.44 | 3.92 | 4.12 |
|  | SD | female | 0.28 | 0.47 | 0.30 | 0.47 | 0.24 | 0.35 | 0.47 |
|  |  | male | 0.66 | 1.23 | 0.94 | 0.94 | 1.47 | 1.68 | 1.51 |
|  |  |  |  |  |  |  |  |  |  |
| ER-like product in gonad-digestive complex | mean | female | 0.87 | 1.17 | 0.66 | 0.86 | 0.98 | 0.74 | 1.21 |
|  |  | male | 2.07 | 1.26 | 1.46 | 1.85 | 1.63 | 1.48 | 1.17 |
|  | SD | female | 0.68 | 0.63 | 0.34 | 0.75 | 0.75 | 0.28 | 0.60 |
|  |  | male | 0.69 | 0.68 | 0.50 | 0.36 | 0.35 | 0.52 | 0.26 |
|  |  |  |  |  |  |  |  |  |  |
| ERR product in cerebral ganglia | mean | female | 2.09 | 1.81 | 1.78 | 2.13 | 1.86 | 1.84 | 2.33 |
|  |  | male | 1.73 | 2.00 | 1.70 | 2.14 | 1.64 | 1.61 | 1.80 |
|  | SD | female | 0.36 | 0.45 | 0.65 | 0.47 | 0.57 | 0.94 | 0.43 |
|  |  | male | 0.79 | 0.71 | 0.46 | 0.28 | 0.70 | 0.43 | 0.40 |
|  |  |  |  |  |  |  |  |  |  |
| ERR product in penis and sheath (M) or albumen gland (F) | mean | female | 0.95 | 0.61 | 0.80 | 0.77 | 0.55 | 0.68 | 0.62 |
|  |  | male | 4.84 | 4.82 | 3.99 | 4.53 | 3.22 | 3.48 | 3.17 |
|  | SD | female | 0.22 | 0.61 | 0.57 | 0.39 | 0.33 | 0.27 | 0.41 |
|  |  | male | 1.28 | 0.81 | 0.49 | 1.35 | 1.33 | 0.61 | 1.52 |
|  |  |  |  |  |  |  |  |  |  |
| ERR product in gonad-digestive complex | mean | female | 0.55 | 0.62 | 0.30 | 0.60 | 0.72 | 0.38 | 0.64 |
|  |  | male | 1.89 | 1.49 | 1.85 | 2.83 | 1.80 | 1.53 | 1.54 |
|  | SD | female | 0.29 | 0.28 | 0.16 | 0.46 | 0.40 | 0.15 | 0.31 |
|  |  | male | 0.85 | 0.97 | 0.60 | 1.02 | 0.57 | 0.74 | 0.79 |
|  |  |  |  |  |  |  |  |  |  |
| Wet.weight  (g) | mean | female | 6.16 | 6.88 | 6.51 | 5.77 | 6.45 | 6.22 | 6.29 |
|  |  | male | 4.93 | 5.13 | 3.81 | 4.27 | 4.66 | 5.02 | 5.02 |
|  | SD | female | 1.18 | 1.62 | 1.45 | 1.20 | 1.23 | 1.25 | 0.58 |
|  |  | male | 0.68 | 1.29 | 0.32 | 0.92 | 0.65 | 0.88 | 1.19 |
|  |  |  |  |  |  |  |  |  |  |
| Shell.height  (mm) | mean | female | 30.70 | 32.20 | 31.70 | 30.29 | 31.13 | 31.32 | 31.53 |
|  |  | male | 28.63 | 29.18 | 26.35 | 27.16 | 28.42 | 28.95 | 28.35 |
|  | SD | female | 2.43 | 2.67 | 2.41 | 2.65 | 2.25 | 1.77 | 0.82 |
|  |  | male | 1.05 | 2.62 | 1.16 | 1.91 | 1.76 | 1.89 | 2.27 |
|  |  |  |  |  |  |  |  |  |  |
| Aperture.width  (mm) | mean | female | 14.92 | 15.44 | 15.40 | 14.87 | 15.48 | 15.32 | 15.53 |
|  |  | male | 14.68 | 14.48 | 13.33 | 13.72 | 14.15 | 14.37 | 14.35 |
|  | SD | female | 0.92 | 1.12 | 0.96 | 0.90 | 1.17 | 0.66 | 0.46 |
|  |  | male | 0.81 | 1.05 | 0.50 | 1.21 | 1.16 | 0.85 | 1.33 |
|  |  |  |  |  |  |  |  |  |  |
| number of snails |  | female | 6 | 7 | 8 | 7 | 6 | 6 | 7 |
|  |  | male | 6 | 5 | 4 | 5 | 6 | 6 | 4 |

Table S3. The mean and standard deviation of the gene expression of two genes in three tissues in females and males and the size of the snails after 12 weeks exposure.

| toxicological  end-point | *p*-value |
| --- | --- |
| ER-like product in cerebral ganglia | 0.89 |
| ER-like product in penis and sheath (M) or albumen gland (F) | 0.71 |
| ER-like product in gonad-digestive complex | 0.95 |
| ERR product in cerebral ganglia | 0.17 |
| ERR product in penis and sheath (M) or albumen gland (F) | 0.21 |
| ERR product in gonad-digestive complex | 0.47 |
| Wet.weight | 0.040 |
| Shell.height | 0.11 |
| Aperture.width | 0.65 |

Table S4. The *p*-value for the hypothesis that the end-point does not affect the number of eggs produced per female during week 6 to 12.

Figure S2. Bar and Whisker plots showing the mRNA expression levels of mcER-like and mcERR genes in the albumin gland assessed by aQPCR at pre-exposure (pre), and after 1 week (wk1), 6 week (wk6), and 12 week (wk12) exposure to 17β-oestradiol (10, 100 and 100 ng/l) , 4-tert-Octylphenol (5 and 25 μg/l) or the water (DW) and solvent controls (SC). Week 1 DW n=6♀ and 5♂; SC n= 7♀ and 5♂; E2 10 n= 8♀ and 4 ♂, E2 100 n= 7♀ and 5 ♂; E2 1000 n= 7♀ and 5♂; OP 5 n=4♀ and 8♂; OP 25 n= 6♀ and 6♂. Week 6 DW n=7♀ and 5♂; SC n= 8♀ and 4♂; E2 10 n= 5♀ and 7 ♂, E2 100 n= 7♀ and 5 ♂; E2 1000 n= 6♀ and 6♂; OP 5 n=7♀ and 4♂; OP 25 n= 6♀ and 6♂ Week 12 DW n=6♀ and 6♂; SC n= 7♀ and 5♂; E2 10 n= 8♀ and 4 ♂, E2 100 n= 7♀ and 5 ♂; E2 1000 n= 6♀ and 6♂; OP 5 n=6♀ and 6♂; OP 25 n= 7♀ and 4♂* show the outliers.

Figure S3. Bar and Whisker plots showing the mRNA expression levels of mcER-like gene in the male and female gonad-digestive complex assessed by aQPCR at pre-exposure (pre), and after 1 week (wk1). 6 week (wk6), and 12 week (wk12) exposure to 17β-oestradiol (10, 100 and 100 ng/l) , 4-tert-Octylphenol (5 and 25 μg/l) or the water (DW) and solvent controls (SC). N=6 snails per time point per treatment. * show the outliers.

Figure S4. Bar and Whisker plots showing the mRNA expression levels of mcERR gene in the male and female gonad-digestive complex assessed by aQPCR at pre-exposure (pre), and after 1 week (wk1). 6 week (wk6), and 12 week (wk12) exposure to 17β-oestradiol (10, 100 and 100 ng/l) , 4-tert-Octylphenol (5 and 25 μg/l) or the water (DW) and solvent controls (SC). N=6 snails per time point per treatment. * show the outliers.

Figure S5. Bar and Whisker plots showing the mRNA expression levels of mcER-like gene in the male and female cerebral ganglia assessed by aQPCR at pre-exposure (pre), and after 1 week (wk1). 6 week (wk6), and 12 week (wk12) exposure to 17β-oestradiol (10, 100 and 100 ng/l) , 4-tert-Octylphenol (5 and 25 μg/l) or the water (DW) and solvent controls (SC). N=6 snails per time point per treatment. * show the outliers.

Figure S6. Bar and Whisker plots showing the mRNA expression levels of mcERR gene in the male and female cerebral ganglia assessed by aQPCR at pre-exposure (pre), and after 1 week (wk1). 6 week (wk6), and 12 week (wk12) exposure to 17β-oestradiol (10, 100 and 100 ng/l) , 4-tert-Octylphenol (5 and 25 μg/l) or the water (DW) and solvent controls (SC). N=6 snails per time point per treatment. * show the outliers.


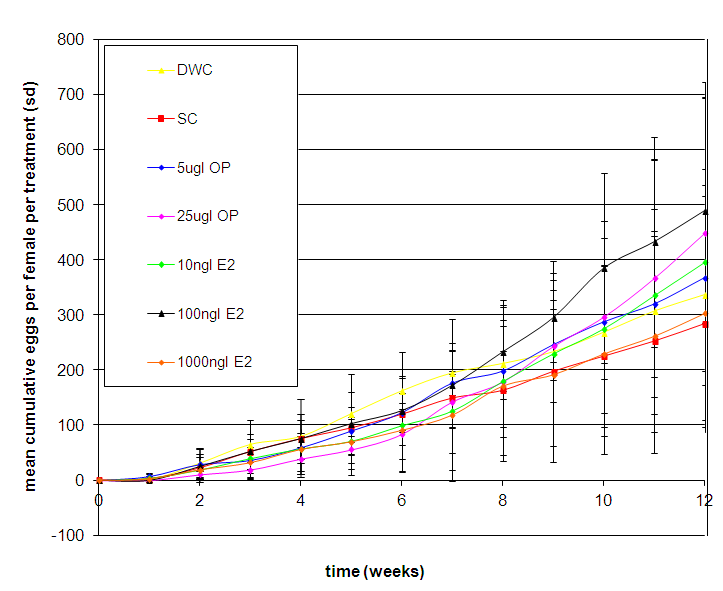


Figure S7. Effect of E2 (10, 100 and 1000 ng/L) and OP (5 and 25 μg/L) on mean cumulative eggs per female per treatment. Values represent the mean ± SD of the three replicate tanks throughout the 12-week exposure.
